# The Epidemiology of Mumps

**Published:** 1915-04

**Authors:** Howard D. King

**Affiliations:** New Orleans, La.


### THT EPIDEMIOLOGY OF MUMPS.

By Howard D. King, M. D., New Orl ans, La.

MUMPS.—An acute infectious and highly contagious disease, characterized by inflammation of the parotid gland, and frequently complicated by orchitis in the male, and mastitis in the female.

In the first place, it is necessary to establish a well marked difference between parotiditis and mumps. This difference is not exclusively based upon the anatomical site of the lesion, since in both conditions the cellular tissue of the parotid gland may be as much involved as the glandular tissue. The difference lies wholly in the nature of the disease itself.

The term parotiditis is applied to inflammations which sometimes supervene during the course or during the decline of severe febrile disturbances and infectious processes, such as scarlet fever, measles, smallpox, typhoid fever, dysentery, diphtheria, puerperal fever and appendicitis. These cases of parotiditis, which often end in suppuration, in necrosis or in gangrene of the gland, are the indication and result of general ill-health, and their appearance is usually of gloomy augury. There are also cases of parotiditis which are associated with stomatitis (mercurial stomatitis), in which the inflammation is purely local, and spreads through Steno's duct to the gland. These inflammations, though of different origins, are due to the ordinary microbes of suppuration, and have nothing in common with mumps.

It may be definitely stated that the causal agent of mumps has not yet been determined. No microscopic or ultra-microscopic parasite has been found in the blood of monkeys subjected to inoculatory experiments; but, during the course of the fever induced as a result of inoculations, the blood count showed a great increase of mononuclear leucocytes. Feeble though the experimental infection may be, yet it is none the less specific, seeing that it protects against a subsequent inoculation. As a matter of fact, in children the parotid may not be sensibly swollen in cases occurring during an epidemic of mumps. The period of incubation in the experimental disease is long and this accords well with that of the natural malady, and the mononuclear leucocytosis of the former agrees with that of the latter.

The period of incubation is from eleven days to three weeks.

One attack of the disease generally confers immunity, but second attacks are fairly frequent.

When does the disease become contagious, and how long does this period of contagiousness last? The disease is especially contagious at the beginning, even before the swelling of the parotid gland has appeared. Contagion is still active during the height of the disease and even during convalescence.

Mumps is most often taken by direct contact through the secretions in the buccal and nasal cavities of persons suffering from the disease. Mumps is hardly ever transmitted a great distance by a third person or inanimate objects. Animals play no part in the transmission of mumps. Instances have been observed in which epidemics were limited by the interposition of a wall, a glass partition, etc. Thus, it appears that the contagious element cannot be conveyed through the air.

Through what channel does the pathogenic microbe enter the human economy? This is rather a vexatious question. Certainly not through the respiratory passages, for the bronchi and lungs are very seldom affected in cases of mumps. The early localization of the disease in the salivary glands speaks in favor of an entrance through the mouth or possibly through the nose by way of the pharynx. It is believed that the casual organism becomes mixed with the saliva and passes up through Steno's duct into the parotid gland. This explanation does not, however, fit well those cases in which the parotid symptoms are delayed and the first manifestation is an orchitis.

Children and adolescents are most frequently attacked by the disease, but adults are by no means exempt from it. Most unfortunately, mumps is too often regarded as a trivial disease, and, thus, the question of age incidence has been neglected. The majority of persons attacked are between the ages of five and fifteen. Infants very rarely suffer, and the disease is uncommon in persons over forty.

In explanation of the rarity of the disease in infants it may be assumed that the infant possesses against mumps a peculiar natural congenital immunity as he does against other infections; or accept the anatomical explanation that the incomplete development of the parotid and the narrowness of its duct offers unfavorable conditions for the infection.

There is a popular notion among medical authorities that mumps do not attack very old people; but this is an error. In epidemics of mumps one frequently sees a member of an infected family who apparently does not have the disease, certainly not in a well defined and distinct manner. But epidemiological inquiry usually reveals the interesting fact that some of these, apparently non-infected, individuals have had some indefinite febrile disturbance during the epidemic. Thus, it would appear, the same as in other diseases, there must be an atypical mumps. Hypothecating the truth of this, will not account for the rarity of the disease in the aged. That there is something in this is evidenced by the fact that in epidemics occurring in isolated regions everyone contracts the disease except infants.

The most notable example of the disease in adults accurred during the War of the Rebellion, when a great number of troops in the field were incapacitated by mumps.

The malady occurs with equal frequency in both sexes. Some epidemiologists, however, assert that it is more commonly met with in boys than girls.

Its relation to race is uncertain; but, in mixed communities the disease frequently displays a selective power attacking persons of one race and sparing others. As to geographical distribution there are no differences discoverable in the incidence of the disease among the several races.

Mumps has a practically universal distribution over the earth's surface. It is true that certain localities may suffer from epidemics of mumps year after year, while others remain free, and that the latter may suddenly and without apparent reason become subject to it.

The disease prefers cold and wet weather to warm and dry, and the majority of epidemics occur in the winter and early spring.

Its prevalence is not dependent on latitude, climate or altitude above the sea level.

Circumstances of soil, hygiene and sanitary conditions have no bearing whatsoever for the origin and spread of mum[illegible]

Institutions, such as boarding schools, orphanages, prisons or barracks, are the usual scenes of epidemics of mumps, but larger communities may be affected. The epidemic as a rule does not strike down a large number of persons at once; it extends by successive outbursts. Mumps usually occurs in small epidemics which are circumscribed to rather limited areas, in which the disease is very severe, though not of long duration. Mumps, in exceptional cases, may become epidemic over a wide area, but has never truly become pandemic.

The disease accidentally introduced into a locality quickly spreads, but it often spends its force in one place without spreading outside. The power of expansion of mumps is not comparable to that of measles, smallpox, whooping cough or influenza. The contagiousness of the malady is undeniable, but it is retsircted. Thus, the accounts of very many epidemics in which the disease was limited wholly to single schools, orphan asylums and garrisons. At times mumps has remained confined to a single regiment; and, again, it would spread to the other troops in garrison while not attacking the civilians of that place, although it may extend to the town outside of the garrison. The study of military epidemics shows that young soldiers are more liable to suffer than veterans.

The frequency of occurrence of epidemics is dependent entirely upon the presence or absence of epidemics. Thus a city may have in one year hundreds or thousands of cases of mumps, and during the next or several successive years there may not be a single case.

Epidemics vary in type from year to year. One year orchitis may be the rule, while in the following year pancreatitis may be the distinguishing complicatory feature of the epidemic. Very often epidemics are masked by the appearance of complications long before the manifestation of the true clinical signs of mumps.

